# Deconvoluting the complexity of autophagy and Parkinson's disease for potential therapeutic purpose

**DOI:** 10.18632/oncotarget.5803

**Published:** 2015-09-22

**Authors:** Jingjing Li, Sijia Li, Lan Zhang, Liang Ouyang, Bo Liu

**Affiliations:** ^1^ State Key Laboratory of Biotherapy and Cancer Center, Collaborative Innovation Center of Biotherapy, West China Hospital, Sichuan University, Chengdu, China; ^2^ State Key Laboratory of Stomatology, West China Hospital of Stomatology, Sichuan University, Chengdu, China

**Keywords:** Parkinson's disease (PD), autophagy, α-synuclein, LRRK2, PD therapy

## Abstract

Parkinson's disease (PD) is a neurodegenerative disorder characterized by the preferential death of dopaminergic neurons. In the past two decades, great progress has been made toward understanding the pathogenesis of PD; however, its precise pathogenesis still remains unclear. Recently, accumulating evidence has suggested that macroautophagy (herein referred to as autophagy) is tightly linked to PD. Dysregulation of autophagic pathways has been observed in the brains of PD patients and in animal models of PD. More importantly, a number of PD-associated proteins, such as α-synuclein, LRRK2, Parkin and PINK1 have been further revealed to be involved in autophagy. Thus, it is now acknowledged that constitutive autophagy is essential for neuronal survival and that dysregulation of autophagy leads to PD. In this review, we focus on summarizing the relationships amongst PD-associated proteins, autophagy and PD. Moreover, we also demonstrate some autophagy-modulating compounds and autophagic microRNAs in PD models, which may provide better promising strategies for potential PD therapy.

## INTRODUCTION

Parkinson's disease is the second most common neurodegenerative disease, which is mainly characterized by the progressive loss of dopaminergic and non-dopaminergic neurons, and the development of intracellular aggregates of the protein α-synuclein. The majority of PD is sporadic, while approximately 5-10% of cases are inherited through PD-related genes [1, 2]. Global disease burden for PD is increasing significantly in recent years, with all ages deaths (thousands) rises by 139.8 (77.36 to 156.99)% from 43.7 (38.3 to 55.1) to 102.5 (79.3 to 112.6) and age-standardized death rate (per 100 000) rises by 28.2% (−0.42 to 37.83) from 1.5 (1.3 to 1.9) to 1.8 (1.4 to 2.0) between 1990 and 2013 [3]. With the growing ageing population, the number of people with PD globally is projected to be at least 8.7-9.3 million by 2030 [4]. In clinical, PD is characterized by the core motor symptoms collectively called parkinsonisms, including resting tremor, bradykinesia, muscle rigidity, postural instability and gait impairment, and it is also accompanied by a range of nonmotor symptoms, including constipation, urinary symptoms, sleep disorder, and dementia [5, 6]. In pathology, PD is featured by progressive degeneration not only within the dopaminergic nigrostriatal system, which leads to loss of dopamine in the striatum and is responsible for the main motor symptoms, but also by implication of a range of other neuronal systems and organs [7]. These lesions are accompanied by widespread occurrence of Lewy bodies (LBs) and Lewy neurites (LNs) composed of abnormal, post-translationally modified, and aggregated form of α-synuclein, the major protein marker and pathological hallmark of PD [6]. Nowadays, multiple methods are applied in PD treatment, including medications, surgery, deep brain stimulation and multidisciplinary management, which substantially provide relief from the symptoms and improve quality of life and functional capacity. However, PD is still an progressive disease with no cure [8].

Recently, increasing evidence suggest that autophagy is closely related to the pathogenesis of PD and investigations on autophagic pathways in PD models may provide new clues on PD therapy [9, 10]. Autophagy is an evolutionarily conserved catabolic process that mediates the degradation of long-lived proteins and dysfunctional or superfluous organelles in cells, and it is induced by various stressful conditions such as limited nutrients, low oxygen levels, and decreased energy supply [11]. In general, autophagy can be divided into three main types: macroautophagy, microautophagy, and chaperone-mediated autophagy (CMA), based on how cargo is delivered to the lysosome. In CMA, chaperone proteins mediate this process by binding to cytosolic substrates that enter the lysosome through interaction with a receptor/channel on the lysosomal membrane [12]. Microautophagy refers to the process of direct uptake of cytoplasmic materials at the lysosome surface by invagination of the lysosome membrane. After vesicles containing the cytosolic substrates pinch off into the lysosomal lumen, they are rapidly degraded [13]. During macroautophagy, portions of the cytoplasm are engulfed by a double-membrane phagophore that expands into a cytosolic vesicle called an autophagosome, and then the autophagosome fuses with a lysosome to form an autolysosome, which degrades the macromolecules [14]. Among the three types of autophagy, macroautophagy is the best characterized process and will be hereafter be referred to as autophagy. Dysregulation of autophagic pathways has been observed in the brains of PD patients and in PD animal models, and a number of PD-associated genes have been reported to impair or induce autophagy in PD [15, 16], indicating the emerging role of autophagy in this disease. In particular, autophagy modulation has become a promising strategy in PD area and identification of targets in autophagy pathways is in urgent need.

## AUTOPHAGY AND PARKINSON'S DISEASE ­(PD)

### Molecular mechanisms of autophagy

Autophagy includes five phases: initiation, elongation, autophagosome formation, fusion, and autolysosome formation. The formation of autophagosomes is initiated in mammalian cells primarily by the Unc51-like kinase 1 (ULK1) complex containing ULK, Atg13, FIP200, and Atg101 [17, 18]. Activation of this complex can be inhibited by mammalian target of rapamycin (mTOR) complex 1, which is a master negative regulator of autophagy in several pathways [19, 20]. Another complex involved in the initiation of autophagy is the class III phosphatidylinositol 3-kinase (PtdIns3K)/Vps34 complex I containing Beclin-1, Atg14 and Vps15 [21]. Elongation and maturation of autophagosomes involves two ubiquitin-like conjugation systems, the microtubule-associated protein 1 light chain 3 (LC3) system and the Atg12 system. Atg12 is conjugated to Atg5 by Atg7 (E1 enzyme) and Atg10 (E2 enzyme). The Atg12-Atg5 heterodimer interacts with Atg16L, and this complex promotes elongation of the autophagic membrane [22, 23]. The full-length LC3 precursor is cleaved by Atg4B, forming LC3I. After autophagy is induced, LC3I is conjugated with phosphatidylethanolamine (PE) by Atg7 (E1 enzyme) and Atg3 (E2 enzyme). PE-conjugated LC3 becomes an insoluble form (LC3-II) that is stably inserted into the autophagosomal membrane [24]. Other proteins, including protein kinase A, AMPK/Snf1 and Pho85 regulate autophagy in response to various types of stress [25].

### Autophagy in PD

Much evidence indicates that autophagy can be involved in PD. First clue for an implication of the autophagy-lysosomal pathway in PD was presented by a pathological study that detected autophagic vacuoles accumulation in the substantia nigra of PD patients [26]. Recent studies demonstrate the association of autophagic signal molecules with α-synuclein pathology in PD, confirming that defects in the autophagy pathway are correlated with neurodegeneration in PD [27, 28]. In additional, inactivation of autophagy by deleting of the essential autophagy gene Atg7 in animal models predisposes to PD-like pathology, further verifying the neuroprotective role of autophagy in the pathogenesis of PD [29]. Furthermore, numerous studies show that the application of autophagy enhancers attenuate PD-related dopaminergic neurodegeneration, both *in vitro* and *in vivo*, supporting the potential therapeutic effects of autophagy modulators in PD treatment [30-32]. Also, treatment of mesenchymal stem cells, which significantly enhances autophagy and impairs α-synuclein expression in cellular and animal models of PD, leads to increased neuronal survival against environmental neurotoxins [33]. Inversely, several other researches also report the possible harmful role of autophagy in PD models. PD related mutants like A53T α-synuclein and G2019S LRRK2 lead to cellular damage via the enhancement of autophagy [34, 35]. Also, some autophagy regulative chemicals such as 6-hydroxydopamine (6-OHDA) and 1-methyl-4-phenyl-1,2,3,6-tetrahydropyridine (MPTP), are correlated with cell toxicity caused by autophagy, which can be ameliorate by autophagy inhibition [36-38]. Collectively, such results strongly suggest that dysregulation of autophagy is related to PD, although whether such dysregulation of autophagy is the cause or the consequence of PD pathology remains unclear and needs more investigation. Based on a number of previous researches, modulating autophagy either by compounds or siRNAs in some PD models in which autophagy was dysregulated can help alleviate the symptoms. Of note, one of those crucial factors result in autophagy dysregulation in PD could be genetic factors, such as SNCA and LRRK2. Mutant or over-expression of such PD-related genes is reported to regulate autophagy either positively or negatively, which may be part of pathogenesis of PD (Table 1). Therefore, it is significant to explore the complexity of these genes/proteins and autophagy, which could shed light on the etiology and therapeutic strategies.

**Table 1:** PD-associated genes/proteins and their roles in autophagy

PARK locus	Gene	Clinical feature	Pathology	Role of autophagy	Reference
PARK1/4	SNCA	Typical PD with common dementia	Lewy bodies	Negative	[39-48]
PARK2	Parkin	Early onset, slowly progressing, usually with sleep benefit	Lewy bodies rarely	Positive	[82-87 ]
PARK5	UCH-L1	Late onset	Unknown	Negative	[104,105]
PARK6	PINK1	Early onset, slowly progressing, usually with sleep benefit	One case with Lewy bodies	Positive	[88-93]
PARK7	DJ-1	Early onset	Unknown	Dual	[97-103]
PARK8	LRRK2	Late onset	Usually Lewy bodies	Dual	[83-87]
PARK9	ATP13A2	Early-onset, with Kufor-Rakeb syndrome	Unknown	Positive	[106-108]

## PD-RELATED GENES/PROTEINS ARE INVOLVED IN AUTOPHAGY MODULATION

### α-synuclein

α-synuclein levels are considered a major determinant of its neurotoxic potential, and cytoplasmic α-synuclein aggregates, referred to as Lewy bodies, are pathological hallmarks of PD [39]. A leading paradigm in PD research suggests that α-synuclein accumulation, resulting from its overexpression or lack of degradation, is one of the important mechanisms causing degeneration of dopaminergic neurons [40]. PARK1- and PARK4-linked PD, which is caused by mutations of wild-type SNCA (α-synuclein) gene, is an autosomal dominant one. PARK1 is caused by missense mutations and PARK4 by multiplications of α-synuclein. Three missense mutations, (A53T, A30P and E46K), in addition to two gene multiplications (duplications, triplications) of the SNCA have been described so far [41-45]. Even sporadic PD cases were reported to be genetically linked to α-synuclein polymorphisms, which modulate α-synuclein transcription [46]. Several post-translational modifications such as phosphorylation, nitration, ubiquitination, oxidation, and dopamine-dependent adduct formation are related to the toxic forms of α-synuclein.

**Figure 1:**
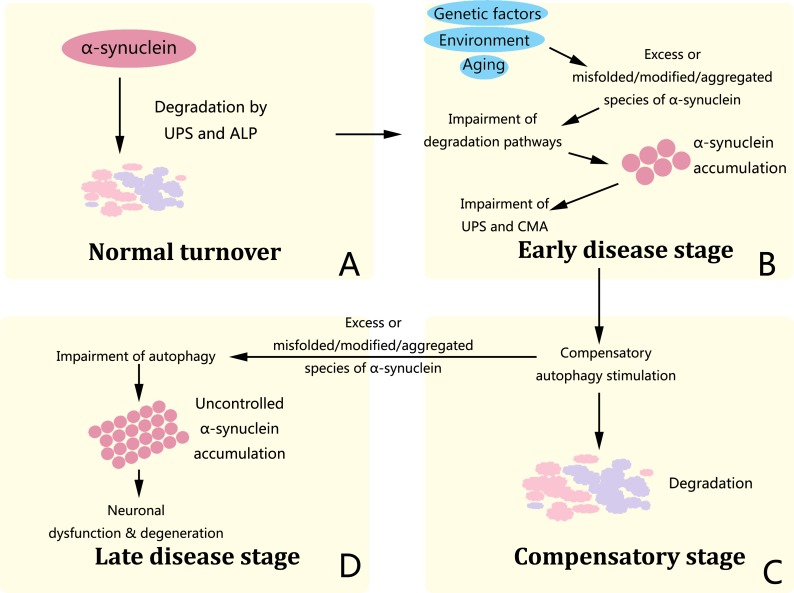
α-synuclein pathology in PD The successive dysfunction of protein degradation pathways is crucial in α-synuclein pathology, neuronal dysfunction and degeneration. **A.** α-synuclein is regularly degraded via both UPS and ALP. **B.** In the early disease stage, primary impairment of degradation pathways induced by genetic, environmental and age-related factors further prevents the degradation of α-synuclein by CMA and UPS and leads to its toxic aggregation in the cytoplasm. **C.** Afterwards, crosstalk among degradation pathways drives the induction of autophagy and temporarily compensate for degradation impairment. **D.** In the late disease stage, α-synuclein accumulation inactivates autophagy, leading to complete dysfunction of all protein degradation pathways and uncontrolled accumulation of α-synuclein, which consequently contributes to neuronal dysfunction and degeneration featured in PD.

α-synuclein levels are closely connected with autophagy in recent studies. α-synuclein occurs natively as a helically folded tetramer, with a great lipid binding capacity, and destabilization of the helically folded tetramer results in α-synuclein misfolding and aggregation found in PD and other human synucleinopathies [47]. α-synuclein is degraded by both ubiquitin-proteasome system (UPS) and the autophagy-lysosomal pathway (ALP), the latter of which consists of CMA and autophagy [48]. Increased levels of α-synuclein or modified forms of the protein prevents its degradation by CMA and UPS, resulting in its toxic aggregation in the cytoplasm and contributing to PD pathogenesis [49, 50]. The autophagy pathway may compensate for the lack of UPS and CMA-mediated degradation and stimulation of autophagy through pharmacological or molecular means leads to increased clearance of α-synuclein and thus neuro-protective, but autophagic cell death may also occur under stressful conditions [51-53]. Importantly, excessive levels of α-synuclein leads to significant inhibition of autophagy, thus a vicious circle is formed and this leads to uncontrolled accumulation of α-synuclein and causes neuronal dysfunction and degeneration (Figure 1) [54-57]. Such evidence indicates that activation of the compensatory way of α-synuclein degradation, i.e. autophagy, to increase clearance of disease-causing proteins thus protecting nerons may be a potent strategy to alleviate PD or other synucleinopathies.

On the other hand, several studies suggest that α-synuclein and its mutants may also influence autophagy modulation in PD, which complicating the issue. Using a transgenic mouse line with human α-synuclein A53T over-expression specifically in dopamine (DA) neurons, researchers uncovered the role of α-synuclein in mitochondria and mitophagy impairments. They observed three clearly defined stages of pathology progression, which indicates that mitochondria and mitophagy impairments might be the cause rather than consequence of neurodegeneration in the DA_SYN53_ mice. α-synuclein may target mitochondria and induces mitochondria-specific macroautophagy (termed mitophagy). Both the immunofluorescence and immuno-EM data support the physical localization of human α-synuclein within mitochondria in DA neurons of the DA_SYN53_ mice [58]. Additionally, it has been reported that α-synuclein contains a cryptic mitochondria targeting signal on its N terminus [59]. It is very likely that α-synuclein may not impair mitochondria functions under normal conditions, but the functional disturbance of mitochondria by α-synuclein may emerge during aging or under pathological conditions [60]. In PD patients carrying α-synuclein gene multiplication mutations, the over-expressed α-synuclein may increase its mitochondria localization and functional disturbance [61]. In the DA_SYN53_ mice, DA neurons exhibit mitochondria fragmentation and prominent intracellular mitochondrial inclusions, but the general autophagy activity is not significantly changed, suggesting that mitophagy is mobilized to specifically remove α-synuclein-damaged mitochondria without affecting general bulk macroautophagy [58]. Inversely, accumulating evidence also suggests that accumulation of A53T α-synuclein impairs autophagy. For instance, A53T α-synuclein over-expression impairs autophagy in SH-SY5Y cells and upregulates mammalian target of rapamycin (mTOR)/p70ribosomal protein S6 kinase (p70S6K) signaling, which is the classical suppressive pathway of autophagy. Silence of mTOR by mTOR siRNA improves the autophagy level and decreases the accumulation of α-synuclein [62]. These evidence show that mTOR/p70S6K signaling plays a critical role in A53T induced disruption of autophagy, making it a possible target for PD therapy. Another mutation in the gene encoding α-synuclein, E46K mutant are also associated with PD. Importantly, it has been reported that the overexpression of E46K mutant α-synuclein impaired autophagy at an early stage of autophagosome formation via the c-Jun N-terminal kinase 1 (JNK1)-Bcl-2 but not the mTOR pathway in PC12 and HEK293 cells transfected with human E46K mutant α-synuclein. Specifically, overexpressed E46K mutant α-synuclein inhibited JNK1 activation, leading to a reduced Bcl-2 phosphorylation and increased association between Bcl-2 and Beclin-1, further disrupting the formation of Beclin-1/hVps34 complex, which is essential for autophagy initiation. In addition, the vulnerability of cells to toxic insults were also increased in overexpressed E46K mutant, which may due to impaired autophagy [63]. Furthermore, other studies demonstrate that although α-synuclein inclusions colocalize with essential components of autophagic pathways, such as p62 and LC3, they cannot be be effectively degraded by autophagy. Results from mammalian cells and transgenic mice suggest that over-expression of α-synuclein compromises autophagy via Rab1a inhibition, which results in the alteration of the autophagy protein Atg9 localization and the inhibition of autophagosome formation [10]. Although the link between α-synuclein and autophagy in PD has not been fully uncovered, such results above demonstrate that autophagy modulation by specific targets in α-synuclein related pathways can be a potent strategy for PD therapy.

### LRRK2

Mutations of PARK8, the gene encoding leucine-rich repeat kinase 2 (LRRK2),are linked to the most common autosomal dominant form of PD, and also some sporadic PD patients [64, 65]. Many LRRK2-related mutations have been reported in PD patients, among which only 6 of these mutations (R1441C/G/H, Y1699C, I2020T and G2019S ) located in the central catalytic ROC-COR-kinase triple domain are clearly pathogenic [66, 67]. Some other LRRK2 variants (G2385R, R1628P) outside of the enzymatic domains represent risk factors for PD, while the significance of other reported substitutions remains unclear [66, 68]. Some evidence suggest that the wild-type LRRK2 protein is degraded by CMA, while the common LRRK2 G2019S mutation impairs this degradation. In addition, a wide variety of studies suggest that LRRK2 is also connected with autophagy. However, the dysregulation of autophagy may be either positive or negative in different mutations and cellular systems. The absence of LRRK2 causes impairment of autophagy-lysosomal pathway, as indicated by accumulation of lipofuscin granules as well as altered levels of autophagy marker proteins LC3-II and p62 [69]. LRRK2 has also been shown to present on autophagosomal membranes isolated from activated macrophage cells, further supporting its regulative role in autophagy [70]. Expression of the R1441C LRRK2 causes impaired autophagic balance, while LRRK2 knockdown increases autophagic activity and prevents cell death induced by autophagy inhibition under starvation conditions [71]. The G2019S mutation was reported to induce striking augmented autophagy in cell experiments, possibly through a mechanism involving excessive mitochondrial fission mediated by dynamin-related protein1 (Drp1) phosphorylation [72-74]. A recent study suggested an effect of the LRRK2 G2019S mutation on induction of mitophagy by interacting with Bcl-2 [75]. Moreover, another study also shows that autophagy deficiency causes increased LRRK2 protein levels and its accumulation in brain [29]. Noticeably, PD patients with LRRK2 mutations frequently exhibit α-synuclein toxicity in the form of Lewy bodies, in which LRRK2 is also present. On the one hand, increased expression of α-synuclein protein has been detected in Y1699C or G2019S LRRK2 PD patient iPS-derived dopamine neurons, indicating the role of α-synuclein in LRRK2 toxicity [76-78]. On the other hand, LRRK2 over-expression enhances α-synuclein-mediated neuropathology by promoting the abnormal aggregation and toxicity of α-synuclein, while α-synuclein cytotoxicity was abrogated by genetic ablation of LRRK2 [79, 80]. The absence of A53T α-synuclein over-expression makes G2019S LRRK2 unable to cause α-synuclein accumulation [80]. However, LRRK2-associated PD is not always characterized by accumulation of α-synuclein. It can be associated with deposition of MAPT/tau protein or ubiquitin-positive inclusions [81]. It was suggested that LRRK2 dysfunction may be upstream to the accumulation of α-synuclein and MAPT, and that other genetic or environmental factors determine which pathology will develop [66]; however, more evidence is needed to support this hypothesis. Still, LRRK2 plays a critical role in pathogenesis of PD and is closely related to autophagy modulation in PD.

### PINK1 and parkin

Mutants in two significant proteins, PINK1 and Parkin, are linked with young onset autosomal recessive PD. Parkin, an E3 ubiquitin ligase implicated in Parkinson's disease is encoded by the 12 exon PARK2 [82, 83]. Over 100 mutations in Parkin have been reported, including missense and nonsense mutations, exonic deletions and multiplications of exons [84-87]. The PARK6 gene contains 8 exons and encodes PINK1, a mitochondrial kinase involved in the mitochondrial dysfunction in the PD pathophysiology [88, 89]. Unlike Parkin, most PINK1 mutations reported are missense and nonsense mutations [72, 90-93].

PINK1 together with Parkin is closely related to mitochondrial function, with PINK1 being upstream of Parkin. The protein sequence of PINK1 reveals a predicted C-terminal kinase domain and a mitochondrial targeting sequence at the N terminus. PINK1 accumulates specifically on damaged mitochondria. PINK1 is degraded rapidly and constitutively in healthy mitochondria, but the degradation of PINK1 is prevented when one mitochondrion becomes impaired, allowing PINK1 to accumulate on the outer membrane of the impaired mitochondrion. After PINK1 accumulation, PINK1 phosphorylates ubiquitin and Parkin to activate Parkin's E3 ligase activity. Parkin is an E3 ubiquitin ligase with an amino-terminal ubiquitin-like domain and a carboxyl-terminal ubiquitin ligase domain. Once activated, Parkin facilitates the mitochondrial clearance via ubiquitylation of specific mitochondrial proteins thereby recruiting autophagic adapters, such as p62 to execute final autophagy [89]. However, mutated forms of PINK1 and Parkin are unable to mediate this process, thus by impairing this process, PD-causing mutations of PINK1 and Parkin may impede mitophagy, cause the accumulation of defective mitochondria, potentially initiate apoptotic events, and, ultimately, lead to neurodegeneration in PD [94].

Moreover, PINK1 is recently reported to be of potential significance in autophagy regulation. By interacting with Beclin-1, PINK1 distinctly enhances both basal and starvation-induced autophagy, which is blocked by Beclin-1 knock down or by inhibition of the Beclin-1 partner Vps34. Conversely, mutant forms of PINK1, are either unable or less effective to promote autophagy, since they lack the ability to interact with Beclin-1 or have defective kinase activity [95]. A recent study demonstrates that PINK1 can interact with, and phosphorylate Bcl-xL, an anti-apoptotic protein of the Bcl-2 family also known to inhibit autophagy through its binding to Beclin-1, thus protecting against apoptotic cell death. However, PINK1-dependent Bcl-xL phosphorylation does not interfere either with the release of Beclin-1 from Bcl-xL or the autophagy pathway in their results [96].

## OTHER PD-ASSOCIATED GENES/PROTEINS IN AUTOPHAGY

Mutations of PARK7/DJ-1 are causes of another young onset autosomal recessive PD. PARK7-linked PD is very rare with its clinical features similar to those of PARK2-linked PD. DJ-1 also implicated in autophagy, with its role still in debate. Early study has shown that loss of DJ-1 resulted in impaired basal autophagy and accumulation of defective mitochondria, which is connected with reduction in phosphorylated ERK2 [97, 98]. Conversely, other studies challenged these results showing that DJ-1 deficiency promotes an enhancement in autophagic activity, as an increase in autophagy markers was observed [99-101]. Another study suggests that DJ-1 may act to alter autophagy indirectly by maintain mitochondrial function during oxidative stress, to be specific, DJ-1 absence reduces NF-κB signaling, which lessen NF-κB-dependent suppression of autophagy via mTOR [102]. Additionally, inhibition of JNK pathway blocks autophagy activation induced by DJ-1 knockdown, indicating that DJ-1 regulates autophagy in a JNK-dependent manner [103].

Mutations in UCH-L1 are associated with PARK5-linked PD, a very rare autosomal dominant PD. Autophagy is proved to be one of the major mechanisms that degrade UCH-L1 [104]. In addition, a recent study shows that UCH-L1 suppression activates the autophagic pathway and thus prevents α-synuclein aggregates formation [105]. PARK9-linked PD represents an autosomal recessive disorder and the gene was identified as ATP13A2, which is also involved in autophagy regulation [106]. ATP13A2 is responsible for α-synuclein clearance via the lysosome, and a failure in this process would lead to the toxic accumulation of α-synuclein in the cytoplasm. Impaired autophagy and accumulation of α-synuclein is observed in both ATP13A2 mutant or deficient cells and elevated ATP13A2 expression leads to reduction of intracellular α-synuclein protein [107, 108]. Mutations of the glucocerebrosidase (GBA) gene represent the greatest genetic risk factor for PD [109]. GBA deficits are correlated with reduced GBA and α-synuclein accumulation, as well as suppression of CMA pathways. However, there is no evidence so far indicating connections between GBA mutations and macroautophagy [110].

## MODULATION OF AUTOPHAGY FOR PD THERAPY

PD is still an incurable disease affecting millions of people worldwide. Many treatment methods, including medications, surgery, deep brain stimulation and multidisciplinary management, are applied in current PD therapy and contribute to better symptom control and life quality [11]. As the etiology of PD remains largely unknown, the available therapeutic approaches of the disease are focused on neuroprotection for cells preservation and symptom control via increasing dopamine levels in involved brain areas. However, the dopamine-based strategy is accompanied by a series of side effects and can be ineffective, due to the possible habituation or dopaminergic neuron death [111]. In view of the increased global disease burden of PD and the deficiency of effective treatments, there is an increased urgency to explore novel therapeutic approaches for PD treatment, one of which is autophagy modulation. From what has been discussed before, it is clear that autophagy play a crucial part in PD, although whether it act as the cause or the result of onset of PD remains unknown. Still, it is worth noting that dysregulation of autophagic pathways has been observed in the brains of PD patients and in PD animal models, which may result from over-expression or mutation of some key PD-related genes, such as SNCA, and modulation of autophagy help contribute to PD remission.

## AUTOPHAGY-MODULATING COMPOUNDS IN POTENTIAL PD THERAPY

A number of compounds have been reported to regulate autophagy in PD models, and several of them relieved symptoms or improved cell viability (Table 2). Extensive researches are done detecting the link between PD and autophagy enhancers, which is suggested as cytoprotective in most results. A natural compound named curcumin, could efficiently reduce the accumulation of A53T α-synuclein through downregulation of the mTOR/p70S6K signaling and recovery of macroautophagy which was suppressed in SH-SY5Y cells [62]. Some other compounds exhibit ability to rescue neurons from toxin-mediated cell death. Rapamycin induces autophagy followed by mTOR/p70S6K inhibition and Bcl-2 induction and shows its neuroprotective ability in lactacystin-induced neurodegeneration, both in *vivo* and in *vitro* [112]. The phenothiazine neuroleptic trifluoperazine activates autophagy in LUHMES neuron and rescues neurons from α-synuclein-mediated cell death [113, 114]. Likewise, two autophagy enhancers, valproic acid (VPA) and carbamazepine (CBZ), strengthened SH-SH5Y survival against rotenone toxicity [30]. Also, trehalose, a novel mTOR-independent autophagy inducer, accelerates the clearance of the A30P and A53T mutants of α-synuclein but not wild type α-synuclein, and also protects neurons from staurosporine-induced cell death [115]. While the protective autophagy role in mentioned compounds above is evidenced by the opposite pro-death effect after autophagy suppression, studies in some other compounds strongly suggest the existence of protective autophagy, though without proving. A Prolyl oligopeptidase (PREP) inhibitor, 4-phenylbutanoyl-L-prolyl-2(S)-cyanopyrrolidine (KYP-2047), enhances autophagic clearance of α-synuclein, both in *vivo* and in *vitro*, via a Beclin-1-dependent pathway [116,117]. The tyrosine kinase Abl inhibitor nilotinib, which is used in adult leukemia therapies, reaches the brain within US Food and Drug Administration approved doses, stimulates autophagic degradation of α-synuclein, protects neurons, increases dopamine levels and thus improves motor performance in mice [118]. Similarly, Latrepirdine ameliorated the degradation of α-synuclein in differentiated SH-SY5Y neurons, and *in vivo* in mouse brains, in parallel with enhanced autophagy [119]. Besides, two glucosylceramide (GlcCer) synthase inhibitors DL-threo-1-Phenyl-2-palmitoylamino-3-morpholino-1-propanol (PPMP) and Genz-123346 (Genz) exert their robust effects on autophagy enhancement in an AKT-mTOR-dependent manner and and reduces mutant α-synuclein levels in neurons. Nonetheless, the pro-survival role of autophagy is not successfully confirmed by autophagy block, indicating that the autophagic degradation does not contribute to the decrease in mutant α-synuclein[120]. Of note, an inhibitor of both wild-type and mutant LRRK2 forms, 2-arylmethyloxy-5-subtitutent-N-arylbenzamide (named GSK257815A), has been reported to induce protective autophagy in dopaminergic cell culture model SH-SY5Y [121].

In other cases, compounds are inversely concerned with cytotoxic mechanisms of autophagy in PD. 6-OHDA, one of the most commonly used neurotoxins for modeling degeneration of dopaminergic neurons in PD, is able to induce autophagic cell death, which can be reverted by pretreated with autophagy inhibition by 3-methyladenine (3-MA) or AKT activation [37, 38]. MPTP also triggers neuronal loss with Cdk-5-associated autophagy activation [36]. The two compounds above are often used for building PD models but not for treatment. Another situation is that compounds may protect cells from death by inhibiting cytotoxic autophagy. For instance, a novel and selective peptide inhibitor Drp1 inhibitor, P110, increases cell viability by reducing autophagic cell death, and reduced loss of primary dopaminergic neurons in a PD model [35, 122].

Together, most evidence support the cytoprotective role of autophagy in PD, suggesting the potential therapeutic value of promoting autophagy by novel compounds, which is the current research focus. Nevertheless, other PD cases may be associated with autophagic cell death, which makes autophagy block another possible strategy for PD. All in all, to protect dopaminergic cells from death via autophagy modulation is the final goal.

**Table 2:** Autophagy-modulating compounds in potential PD therapy

	Compound	Structure	Target	Autophagic Pathway	Cell Type/Animal strain	Autophagic role	Reference
**Autophagy inducer**	Trifluoperazine	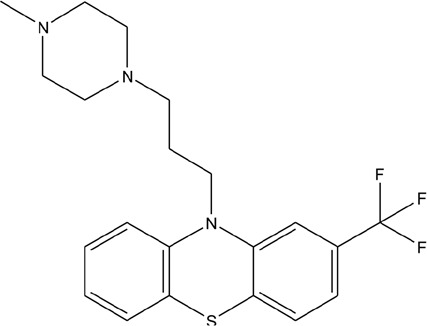	Calmodulin	Unknown	LUHMES cells	Pro-survival	[113,114]
	Rapamycin	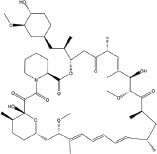	mTOR	mTOR/p70S6K inhibition and Bcl-2 induction	PC12 cells andC57BL/6 mice	Pro-survival	[112]
	VPA	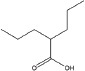	Unknown	Unknown	SH-SY5Y cells	Pro-survival	[30]
	CBZ	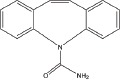					
	Trehalose	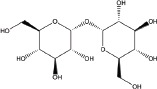	Unknown	Unkown but mTOR independent	SKN-SH cells	Pro-survival	[115]
	6-OHDA	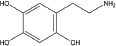	Cathepsin L	Unknown	C57BL/6 mice	Pro-death	[38]
					Sprague–Dawley rats		[37]
	MPTP	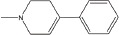	Cdk5	Interaction with UVRAG/Beclin 1 complex	C57BL/6 mice	Pro-death	[21]
	Latrepirdine	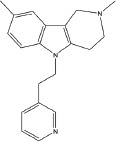	Unknown	Unknown	SH-SY5Y cells and C3H/He-C57BL/6 mice	Not evidenced	[119]
	KYP-2047	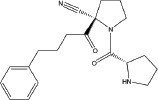	PREP	Beclin-1 overexpression	C57BL/6 mice	Not evidenced	[116]
					Neuro-2A cells		[117]
	Nilotinib	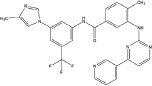	Abl	Beclin-1 overexpression	C57BL/6 mice	Not evidenced	[118]
	PPMP	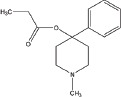	GlcCer synthase	AKT-mTOR Inhibition	HEK293 EGFP-p62 cells	Not evidenced	[120]
	Genz-123346	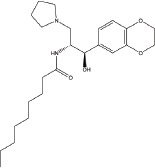					
	GSK257815A	Unavailable	LRRK2	Unknown	SH-SY5Y cells	Pro-survival	[121]
**Autophagy inhibitor**	P110	Unavailable	Drp1	Drp1 inhibition	HEK293T and LRRK2 G2019S-iPS cells	Pro-death	[35]
					SH-SY5Y cells		[122]

## AUTOPHAGY-MODULATING MICRORNAS IN POTENTIAL PD THERAPY

MicroRNAs (miRNAs), small and non-coding RNA molecules of 22 ∼ 24 nucleotides in length, are involved in a number of homeostatic processes, including cell survival, proliferation, apoptosis and autophagy [123, 124]. Several studies have provided links between miRNAs and autophagy modulation. For instance, miR-181A can regulate starvation- and rapamycin-induced autophagy through targeting of ATG5 in MCF-7, Huh-7 and K562 cells [125], and miR-376b controls starvation and mTOR inhibition-related autophagy by targeting ATG4C and Beclin-1 in MCF-7 cells [126]. Furthermore, miRNAs are pathologically altered during the inexorable course of PD, suggesting that miRNAs may also be contributing factors in PD [127,128]. And according to recent studies, the relationships between miRNAs and autophagy in PD have been explored. It is reported that miR-595 positively regulates ULK1 thereby inducing autophagy in SH-SY5Y cells, while miR-4487 negatively regulates ULK1 and thus inhibiting autophagy [129], which indicates that autophagy-modulating miRNAs in PD can be explored for potential PD therapeutic purpose. However, investigations on autophagy-modulating miRNAs in PD is just beginning, and some important questions remain to be addressed. Although the identification and validation of miRNA targets has greatly improved, we know little regarding the cellular and molecular circuits in which they are involved. Moreover, there may be a complicated regulatory network of multiple miRNAs and multiple downstream genes. Up to now, studies on miRNAs in PD is inadequate, and greater attention should be paid to autophagy modulation via miRNAs for potential PD therapy.

**Figure 2:**
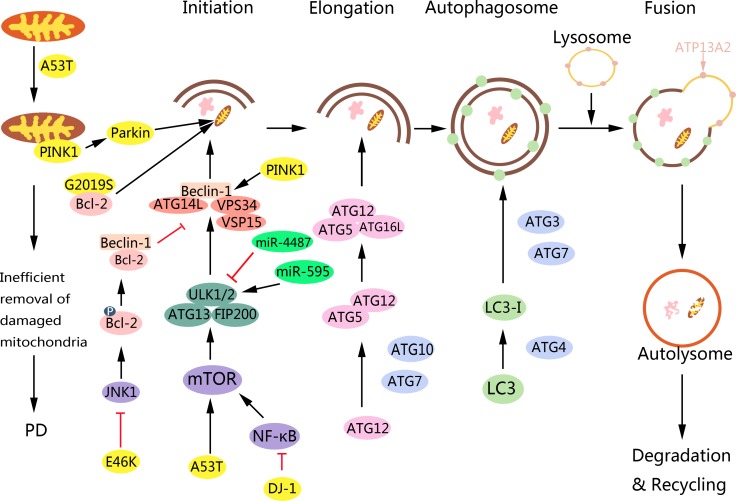
Role of PD-associated proteins in autophagy and targeting autophagy pathway for PD therapy During aging or under pathological conditions, α-synuclein A53T can cause mitochondria damage and induce mitophagy. However, damaged mitochondria cannot be removed efficiently through mitophagy, which contributes to PD. Also, A53T impairs autophagy in SH-SY5Y cells by upregulating mTOR signaling. E46K α-synuclein impairs autophagy via JNK1-Bcl-2 but not the mTOR pathway in PC12 and HEK293 cells. LRRK2 can regulate autophagy either positively or negatively, while its molecular mechanism still remains unclear. According to a recent study, LRRK2 G2019S mutation can induce mitophagy by interacting with Bcl-2. Also, LRRK2 dysfunction may be upstream to α-synuclein. PINK1 or parkin is indispensable for the proper autophagic removal of damaged mitochondria. PINK1 accumulates specifically on damaged mitochondria. After PINK1 accumulation, PINK1 phosphorylates ubiquitin and Parkin to activate Parkin's E3 ligase activity. Once activated, Parkin facilitates the mitochondrial clearance via ubiquitylation of specific mitochondrial proteins thereby recruiting autophagic adapters, such as p62 to execute final autophagy. Mutations of PINK1 and Parkin may impede mitophagy, cause the accumulation of defective mitochondria, potentially initiate apoptotic events, and ultimately lead to neurodegeneration in PD. In addition, PINK1 can enhance autophagy by interacting with Beclin-1. DJ-1 can alter autophagy via NF-κB signaling. ATP13A2 is responsible for α-synuclein clearance via the lysosome, and a failure in this process would lead to the toxic accumulation of α-synuclein in the cytoplasm. miR-595 positively regulates ULK1 thereby inducing autophagy in SH-SY5Y cells, while miR-4487 negatively regulates ULK1 and thus inhibiting autophagy, which may provide potential strategies for PD therapy.

## CONCLUSIONS

PD is one of the most frequent neurodegenerative movement disorders in the world, and many treatment such as medications and surgery are applied in current PD therapy. Modulation of autophagy represent one of novel therapeutic approaches for PD. Recently, it is reported that autophagy plays a pivotal role in the pathogenesis of PD and many PD related proteins are implicated in the regulation of autophagy pathway, such as α-synuclein, LRRK2, PINK1 and Parkin. In addition, a number of compounds have been found to alleviate the symptoms of PD via modulation of autophagy. Nevertheless, information about components involved in autophagy in PD remains limited and functions of some autophagy related components were not confirmed in PD models, which restrains PD therapy targeting autophagy. Currently, most reported compounds that regulate autophagy in PD models target unknown proteins or unknown pathways and specific targets in PD still lack now. Remarkably, it is likely that crucial regulators of autophagy, such as ULK1 and Beclin-1, can be targets for PD drug discovery. It has already been reported that two miRNAs target ULK1 in SH-SY5Y cells and induce or inhibit autophagy. In particular, using new techniques like genomics and proteomics, more new markers and targets in PD will be found, which may shed light on pathogenesis of PD, and also help find potent drugs targeting autophagy for potential therapeutic purpose.
